# A Scoping Review of African Health Histories from the Pre-Colonial to SDG Eras: Insights for Future Health Systems

**DOI:** 10.3390/healthcare14020147

**Published:** 2026-01-07

**Authors:** Humphrey Karamagi, Chinwe Iwu-Jaja, Akhona V. Mazingisa, Abdu A. Adamu, Elizabeth O. Oduwole, Anabay Mamo, Sokona Sy, Charles S. Wiysonge

**Affiliations:** 1 World Health Organization Regional Office for Africa, Brazzaville P.O. Box 06, Congo; karamagih@who.int (H.K.); abdu.adamu@gmail.com (A.A.A.); amamo@who.int (A.M.); ssy@who.int (S.S.); charles.wiysonge@mrc.ac.za (C.S.W.); 2 Department of Community Health Studies, Faculty of Health Sciences, Durban University of Technology, Durban 4000, South Africa; akhonamazingisa04@gmail.com; 3 Vaccines for Africa Initiative, School of Public Health, University of Cape Town, Cape Town 7925, South Africa; oduwoleelizabeth@gmail.com; 4 Cochrane South Africa, South African Medical Research Council, Cape Town 7500, South Africa; 5 Department of Global Health, Stellenbosch University, Cape Town 7505, South Africa

**Keywords:** African health histories, health systems, colonialism, post-independence, primary health care, millennium development goals, sustainable development goals, universal health coverage, Africa

## Abstract

**Highlights:**

**What are the main findings?**

- This scoping review identified 83 records spanning six historical periods (pre-colonial to Sustainable Development Goals [SDG] eras) across 21 African countries, revealing how health systems evolved from traditional community-driven practices to Western-influenced models with persistent tensions between indigenous and colonial medical approaches.
- African health systems demonstrated advanced medical practices even in pre-colonial times, challenging stereotypes, while colonial-period marginalization of traditional medicine created structural inequities and workforce hierarchies that continue to impact contemporary health care delivery.

**What is the implication of the main finding?**

- Historical understanding is essential for designing equitable health systems that address colonial legacies and structural barriers rather than reproducing past inequities, particularly as African countries develop universal health coverage frameworks in the SDG era.
- Significant documentation gaps exist, especially for pre-colonial periods and many African countries, necessitating standardized reporting guidelines for health historical research, centralized repositories, and integration of oral histories to preserve valuable knowledge for future health system strengthening.

**Abstract:**

**Background:** This scoping review aims to systematically examine the extent of the literature on African health histories throughout the pre-colonial, colonial, post-independence, primary health care (PHC), Millennium Development Goals (MDG), and Sustainable Development Goals (SDG) periods. **Methods:** This scoping review followed the Arksey and O’Malley framework, enhanced by Levac’s updates and adaptations from the Joanna Briggs Institute’s methodology. Data from eligible records were extracted based on inclusion criteria and summarized narratively. **Results:** We included 83 records, of which the majority (n = 70) were narrative reviews. Eighteen of these provide evidence from Africa as a whole, while country-specific evidence was obtained from 21 countries. South Africa had the most records (n = 17), followed by Ghana (n = 6) and Nigeria (n = 6). The majority of evidence came from the colonial period (n = 13), followed by the PHC and MDG periods (n = 12 each). Health systems in the pre-colonial era were rooted in indigenous practices and community-driven systems; the colonial period introduced Western-style health care systems; the post-independence period focused on health promotion initiatives and infectious disease eradication; the PHC era focused on community-centered health care and equitable service provision; the MDG era expanded on targeted interventions for infectious diseases, and the SDG era aims to build resilient and inclusive health care systems towards universal health coverage. **Conclusion:** This review revealed diverse influences on health systems from the pre-colonial to SDG eras. However, these records are not exhaustive and require country-specific records, archived documents, and a centralized repository. Addressing these gaps will provide a comprehensive understanding of African health histories and aid in future health interventions.

## 1. Introduction

African health care systems have had a complicated past that includes traditional practices, colonial influences, efforts made after independence, and current global health initiatives [1], and the value of incorporating historical perspectives into public health discourse cannot be overemphasized. Understanding the evolution of these health systems requires a comprehensive examination of the historical contexts that have shaped health practices, health care delivery, and health policies across the continent [1].

Drawing on existing historical analyses of African medicine and public health, we identify six broad periods through which health practices and health care delivery systems on the continent have evolved. These periods provide a conceptual lens for understanding the major transitions that have shaped African health systems over time. The pre-colonial period (around the 1800s in many countries) was characterized by health care practices before contact with settlers, where medicine was purely traditional [2]. This was followed by the colonial period (up to autonomy in some countries), during which colonizers interacted with local communities and introduced different medical approaches, such as Western or Eastern medicine practices [3]. Next came the period immediately following independence (corresponding roughly to the 1960s in some countries), when local autonomy was reintroduced, and indigenous people were responsible for health decision-making. Subsequently, the structural adjustment program, also known as the Primary Health Care (PHC) period (corresponding to the 1970s–1990s) [4], emphasized that ‘Governments have a responsibility for the health of their people by providing a set of basic services needed by the majority of the population.’ The Millenium Development Goals (MDG) period (corresponding to 2000–2015) then focused on delivering a suite of interventions targeting the biggest contributors to morbidity and mortality [5]. Finally, the Sustainable Development Goals (SDG) period (post–2015) shifted the focus to providing a range of interventions targeting the health and well-being of people at all ages [6].

There has been a lack of attention paid to the history of health systems in African countries [7]. This scoping review therefore aims to systematically examine the extent of the literature on African health histories from the pre-colonial to the SDG period. By documenting and understanding these historical trajectories, we can gain valuable insights into the areas where evidence is strong and where gaps exist, as well as better contextualize current health challenges and leverage lessons from the past to shape more equitable and effective health care systems moving forward [7].

## 2. Materials and Methods

This review followed the framework for conducting scoping reviews outlined by Arksey and O’Malley [8] and updated by Levac et al. [9], with adaptations from the Joanna Briggs Institute (JBI) [10,11]. A protocol for this review has also been published previously with detailed methodology [1] and this review has been registered with the Open Science Framework (osf.io/avwut). The steps followed in this review include “defining the research question”, “identifying relevant studies”, “selecting studies”, “charting the data”, and “collating, summarizing, and reporting the results” [8]. We reported this review in accordance with the Preferred Reporting Items for Systematic Reviews and Meta-Analyses extension for scoping reviews (PRISMA-ScR) [12].

### 2.1. Defining the Research Question

As defined in the protocol for this review [1], the research question is “what literature exists on the history of health practices and health care delivery systems in Africa from the pre-colonial era through to the SDG era”?

### 2.2. Identification of Relevant Studies

We conducted a comprehensive literature search in the PubMed database using relevant keywords with the search strategy detailed in Table A1. Additionally, we searched the Google Scholar and Google search engines for gray literature. Due to time constraints, we limited our search to these databases and search engines, which represented a slight deviation from the protocol’s plan to search more databases. The first 100 results each from both Google and Google Scholar were retrieved for screening. We screened articles directly on these search engines, and did not merge them with those from PubMed; therefore, deduplication of articles was not conducted.

### 2.3. Inclusion and Exclusion Criteria

We used the JBI’s Population, Concept, and Context (PCC) framework [10,11] to determine studies eligible for inclusion. The population that we referred to was the African population, such as health care providers, policymakers, and communities. The concept focused on the evolution of health care practices and systems in Africa. This was key to understanding how health systems in Africa transformed over time. The context was both geographical and time-based. The geographical context was Africa as a continent, including sub-regions and countries. The time-based context comprised six distinct time blocks: the pre-colonial, colonial, postcolonial, PHC, MDG, and SDG periods. Studies were assigned to these time blocks based on the historical period they described, rather than their publication dates, as some records examined earlier or overlapping eras.

We included any study that discussed any aspect of health practices and health systems employed and/or developed during any of the specified periods in a historical context. No restrictions were applied regarding the year of publication, language, or study design. For publications in languages other than English, we used Google Translate for translation. Publications that did not pertain to Africa or African populations were excluded. Likewise, studies were excluded if, despite matching search keywords, they focused primarily on biomedical mechanisms, experimental research, or sectoral issues such as biodiversity conservation or regulation, without examining health practices or health care delivery systems. Studies focusing solely on epidemiological analyses of disease burden, trends, or risk factors without examining health practices or health care delivery systems were excluded.

### 2.4. Study Selection

Following the search, all identified records were imported into the Rayyan web application for conducting reviews [13]. Two authors (CJI and AVM) screened both titles and abstracts of these records to obtain potentially eligible ones. The full texts of these potentially relevant records were further assessed by the two authors (CJI and AVM) for eligibility based on the inclusion and exclusion criteria. We resolved any conflicts through discussion and consensus.

### 2.5. Charting the Data

We extracted data from all included studies using a pre-tested extraction tool designed in an Excel spreadsheet. The information collected from each record included study identification (ID), which consisted of the first author’s last name; the year of publication; and the historical year, that is, the period upon which the historical context in the record was based and the corresponding time block (derived from the historical year).

### 2.6. Collating, Summarizing, and Reporting Results

**Figure 1:**
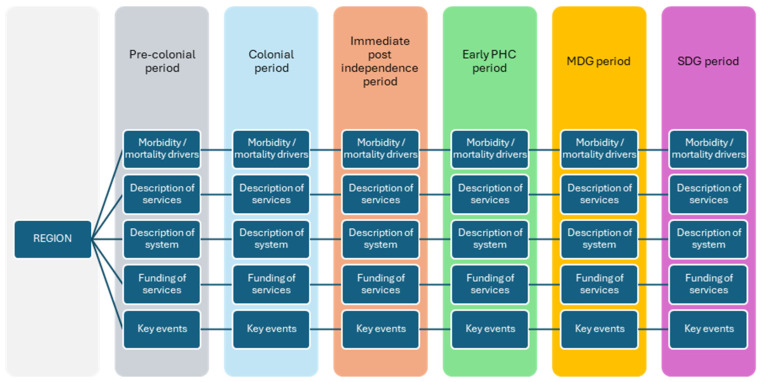
Conceptual framework for documenting African health histories in Africa.

We analyzed the relevant information extracted from each paper and presented them in tables. Given the highly heterogeneous nature of the scoping review and the fact that a meta-analysis was not required, we synthesized the evidence narratively, categorizing our key findings according to each time block. We developed a conceptual framework (Figure 1) to guide our synthesis of the evidence. In this framework, “region” refers to the African region, and the repeated categories represent consistent analytical domains applied to each time block to examine how health practices and health care delivery systems evolved over time. During data extraction and synthesis, information from the included studies was grouped by historical time block. As an extension of this synthesis, recurring elements described across studies were identified inductively and later summarized into three cross-cutting domains, namely, morbidity and mortality drivers, description of health services and systems, and key historical or policy events to support comparative interpretation across periods.

## 3. Results

### 3.1. Results of Literature Search

**Figure 2:**
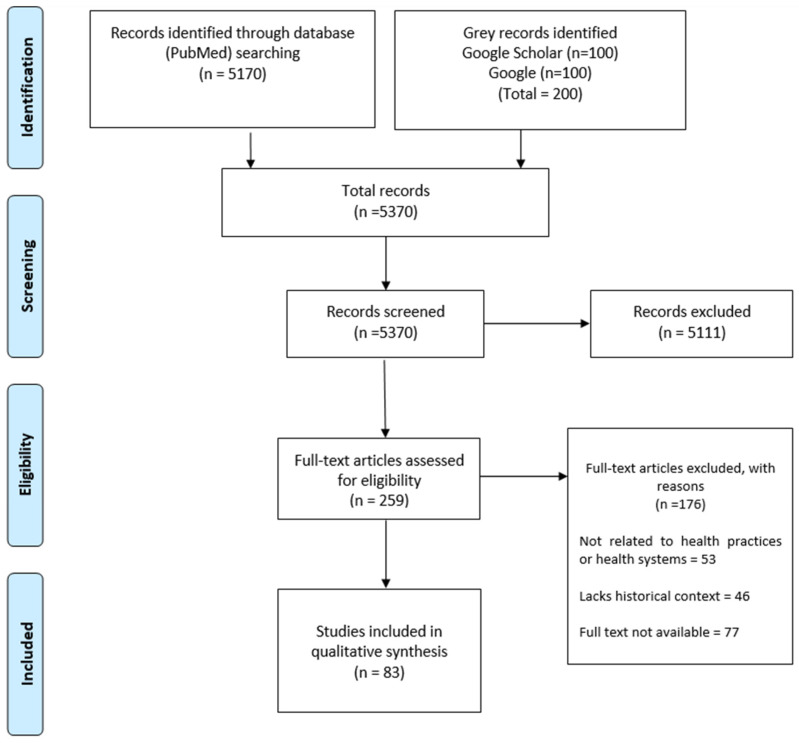
PRISMA flow diagram illustrating the study selection process.

Our literature search initially identified 5371 records. After screening titles and abstracts, 5111 records were excluded. We then reviewed the full texts of the remaining 259 potentially eligible records, from which 176 were excluded. Ultimately, 83 articles were included in this review. Figure 2 below provides the PRISMA flow diagram detailing the study selection process.

### 3.2. Characteristics of Included Studies

The geographical distribution of included studies (Table A2) demonstrates that the majority of the studies (n = 18) focused on Africa as a whole. At the country level, we found studies from 21 countries, with South Africa having the highest number of studies (n = 17), followed by Ghana (n = 6) and Nigeria (n = 6). In comparison, fewer studies focused on sub-regions such as West and Central Africa (n = 2), and one study each was from Central Africa, East and Central Africa and Southern Africa. These records were predominantly narrative reviews (70); there were also fewer systematic reviews (n = 3) and historical analyses (n = 3). The remaining records consisted of one study each for archival research, descriptive studies, document analysis, mixed methods studies, case studies, observational studies, and qualitative studies (Table A2).

The distribution of studies by time block (Table 1) indicates that most of the included studies were published during the colonial (n = 13), PHC (n = 12), and MDG (n = 12) periods, with fewer studies from the pre-colonial (n = 4) period. We also identified studies with historical contexts spanning multiple periods, such as from the colonial to PHC (n = 8), pre-colonial to SDG (n = 1), and post-independence to SDG (n = 2) (Table A2).

### 3.3. Summary of Evidence on African Health Histories Across All Time Blocks

#### 3.3.1. The Pre-Colonial Period

The four studies that were found to explore the historical context during this era focused on the history of medicine in Egypt by linking it to the biography of a physician-king named “Aha.” [14]; the interpretation of diseases, particularly smallpox, in pre-colonial Dahomey (now the Republic of Benin) [15]; insight into the evolution of medical knowledge in ancient Egypt, citing the Edwin Smith papyrus and drawing connections to modern clinical methods [16]; and traditional public health practices in Africa before the 20th century, highlighting the role of kings, chiefs, and priests in managing health through practices like rainmaking and sorcery control [2] (Table A2).

#### 3.3.2. Colonial Period

Thirteen studies covered the colonial period [17,18,19,20,21,22,23,24,25,26,27,28,29]. Six of these studies focused on the influences of the colonial era on different aspects of the health system. These included colonial influences on traditional or indigenous health care systems, including the marginalization of traditional medical practices, the role of traditional leaders in promoting colonial health care and preserving indigenous medical practices, and the decolonization of global health [18,19,20,23,26]. Additionally, their influence on health promotion, including those on nutritional practices [27,28], was noted (Table A2).

Three studies on the colonial era focused on the history of endemic diseases, disease outbreaks and epidemics including onchocerciasis (river blindness) and sleeping sickness, and the roles played in disease control, and also history challenging the misconception that Africans had no role in biomedicine, highlighting their contributions to global health [21,22,25]. Two studies focused on surgical practices at the time, with one reporting that advanced surgical practices were already documented in this era in Uganda, which were at par with Western medicine at the time [24], and another discussing thoracic surgery, which first began in South Africa and moved to other African countries [29] (Table A2).

#### 3.3.3. Post-Independence Period

Five studies described the historical context during this period [30,31,32,33]. The historical development of health promotion in Namibia was discussed in one study [30]. Another article explored the history of naturopathy in South Africa [31]. A separate paper provided a historical perspective on the successful eradication of smallpox and control of measles in West and Central Africa and how these achievements influenced the development of health systems [32]. Additionally, a study examined the 1960s health planning efforts by the WHO, in collaboration with the United States Agency for International Development (USAID), in West and Central Africa, highlighting challenges related to administrative capacity, financial resources, and the implementation of health plans [34]. Lastly, one paper looked at the evolution of dentistry in Uganda and the devastating impact of two decades of civil strife on health care, offering insights into the poor performance of the oral health system [33] (Table A2).

#### 3.3.4. Primary Health Care Period

There were 12 articles in this category [35,36,37,38,39,40,41,42,43,44,45,46]. The historical context discussed in these papers focused on various themes, namely, workforce training to improve health services and address human resource gaps such as training of traditional birth attendants [35], the design and implementation of workforce schemes such as the Return of Service (RoS) scheme in Southern Africa [40], and the African Field Epidemiology Network [41]. Topics related to infectious diseases, including epidemics and control, were highlighted [36], such as the development of the Onchocerciasis Control Programme in West Africa [37] and the HIV/AIDS epidemic and its history in Africa [38] alongside nationwide HIV initiatives for counseling and testing in Rwanda [39]. Other papers focused on occupational health in South Africa [42], Rwanda’s post-genocide health system development [43], the evolving role of traditional medicine in African health systems [44], and government-driven family planning services aimed at managing population growth and improving health through primary care [45,46] (Table A2).

#### 3.3.5. The Millennium Development Goal Period

Twelve studies were included in this category [47,48,49,50,51,52,53,54,55,56,57,58]. The health histories during this period were found in 12 studies. Two studies from South Africa explored the history and post-apartheid reforms of components of their health system and how some of the public health challenges during the MDG period were linked to these reforms and policies [48,50]. Similarly, two other studies from Seychelles and Somalia highlighted the history and evolution of their health systems, including challenges faced in each country [55,56] (Table A2).

Papers that described the response to diseases and outbreaks were also found. One of them provided a review of the history and public health response to Ebola outbreaks across the continent [49], while another one focused on the history of the response to the first Ebola case in Senegal [47]. Similarly, for papers related to disease control, we found the history of the establishment and rollout of a novel initiative to prevent mother-to-child transmission of HIV (Option B+ strategy) and its influence on the country’s health system in Malawi [51] (Table A2).

Two papers provided highlights of historical perspectives of the establishment of various surveillance systems such as the MenAfriNet consortium for meningitis surveillance in Western African countries [54] and the implementation of the Global Antimicrobial Resistance and Use Surveillance System (GLASS), which includes the development of national antimicrobial resistance (AMR) surveillance systems and the reporting of AMR data by African countries [58] (Table A2).

Other studies also provided historical perspectives on other key initiatives such as the Meningitis Vaccine Project (MVP) in Africa, which focused on efforts to develop and introduce a group A meningococcal conjugate vaccine in Africa [57]; a historical overview of family medicine in Malawi highlighting the evolution of training and practice in this field [52]; and the inception of the General Practitioner Contracting Initiative (GPCI) in South Africa, a health financing initiative that aims to achieve universal health coverage [53] (Table A2).

#### 3.3.6. The Sustainable Development Goals Period

The five studies from this period focused on the following: Health financing initiatives aimed at achieving universal health coverage, such as the national comprehensive multi-year strategic plans (cMYP) processes for immunization [59] as well as the International Monetary Fund’s austerity measures for Mozambique’s health system [60]. The history of the evolution of faith-based organizations in helping Ghana realize UHC [61] was also explored. Lastly, a study describing the history of a new community health care worker program (which was initially part of the PHC period) aimed at strengthening Zambia’s health system was found in this category [62] (Table A2).

#### 3.3.7. Cross-Cutting Studies Spanning Different Periods

##### Colonial to PHC Period

Eight studies spanned between the colonial and PHC eras [63,64,65,66,67,68,69,70]. They mostly focused on the evolution of surveillance systems [63]; the evolution of health systems from the colonial era to the PHC era, including challenges due to colonial systems, such as the segregationist approach of the apartheid system in South Africa [64,66,68]; the contributions of missionary organizations [65,69]; and establishment of task-oriented practices among nurses [67] and initiatives to improve maternal and child outcomes [70] (Table A2).

##### PHC to MDG Period

The five articles in this category primarily focused on the evolution of the PHC in countries until the MDG period [71,72,73,74,75]. One study examined the evolution of the PHC in the context of the PHC HIV epidemic in Africa [71], while others focused on the establishment of prevention of mother-to-child transmission (PMTCT) programs and tuberculosis response initiatives [72,73,74]. In addition, one study discussed a special initiative designed to improve PHC services [75] (Table A2).

##### Pre-Colonial to Colonial Period

Of the two studies we found in this cross-cutting era, one study described a historical account of disease control and public health practices in Africa from the pre-colonial to colonial era, specifically focusing on interactions between indigenous and colonial medical systems [76]. The second study went on to describe, on the country level, infectious diseases that have affected Madagascar and surrounding islands over the centuries, such as smallpox, cholera, and Bubonic plague, as well as their response, the establishment of the Pasteur Institute, and vaccine development efforts [77] (Table A2).

##### Pre-Colonial to MDG Period

Only one study was found to describe the history between the pre-colonial and MDG eras. This study provided a historical analysis of the public health landscape in South Africa, while highlighting the impact of social and environmental factors on public health, along with the significant role of HIV/AIDS in shaping the country’s health outcomes [78] (Table A2).

##### Pre-Colonial to SDG Period

Two studies were found to describe health history from the pre-colonial to the SDG era. One study provided a historical overview of the challenges and successes in responding to disease outbreaks in Nigeria, focusing on outbreaks like Yellow fever, Poliomyelitis, Lassa fever, Ebola virus disease, and mpox [79]. The second record was a comprehensive report from Uganda, providing a breakdown of historical events that occurred in each time block [80]. The pre-colonial period was characterized by traditional and complementary medicine, the use of herbal remedies, and traditional birth attendants, making health care very affordable. The colonial era saw the “criminalization” of traditional practices and introduction of Western-style services, including the establishment of missionary hospitals. However, both traditional and Western medical services continued to coexist. The post-independence period was marked by an increase in Western health services within the country, which were regarded as the best in Africa at the time, though this was unfortunately cut short by civil wars that lasted up to 20 years. By the MDG era, health care services in Uganda had not fully recovered from the challenges of the post-independence period. There were health system challenges such as poor infrastructure, out-of-pocket payments, low availability of medicines, and human resource challenges such as staff shortages and lack of payment. There was a gradual introduction of Eastern medicine, particularly from China, in addition to traditional and Western medicine. Health financing was mostly in-kind or trade by barter during the pre-colonial era, by the colonial (British) government during the colonial era, and by the Ugandan government during the post-independence period [80] (Table A2).

##### Colonial to MDG

The seven studies which we identified during this cross-cutting era explored the following: a review of Rift Valley fever (RVF) outbreaks in eastern Africa [81]; a comprehensive historical analysis of South African medicine, touching on colonial and post-colonial influences [82]; schistosomiasis control strategies [83]; and the impact of historical factors such as armed conflicts in former French colonies, particularly Côte d’Ivoire, on health systems [84]. The famous Pholela experiment in South Africa was also discussed for its significant role in the evolution of primary health care [85]. Additionally, the evolution of Nigeria’s health care system from colonial times to the SDG era was examined [86], as well as the role of the private health sector in complementing the public system in South Africa, particularly in health care financing and efforts toward universal health coverage [87] (Table A2).

##### Colonial to SDG Period

Three studies were found to describe histories that cut across the colonial to the SDG eras [88,89,90]. These studies explored the historical development and implementation of PHC in Tanzania, drawing connections to contemporary debates on universal health coverage (UHC) [88]; the history of Rwanda’s malaria control efforts, emphasizing the factors driving change and future strategies in combating the disease [89], and the evolution of health financing reforms in South Africa from the 1920s to 2019, exploring how socio-political factors have influenced these reforms at various points in time [90] (Table A2).

##### Post-Independence to PHC Period

We found two papers that reviewed the development of mental health policy, especially on how mental health practices evolved within the broader health system during these eras [91] and the training of health workers to improve service delivery [92] (Table A2).

##### Post-Independence to SDG Period

We included two papers in this category [92,93]. These included efforts to improve the primary health care system, including efforts in workforce training [92], and challenges in promoting local pharmaceutical production over time. The latter highlights the evolution of policies and initiatives aimed at reducing reliance on imports and enhancing domestic production of health products [93] (Table A2).

##### MDG to SDG Period

We included two studies that described the historical background of the Uganda National Institute of Public Health (UNIPH) and lessons learned so far [94] and the creation of an initiative, “Community-based Health Planning and Services (CHPS)”, to address barriers to access to quality care [95] (Table A2).

Table 1 provides a cross-period summary of key recurring elements identified across the six historical time blocks, highlighting reported morbidity and mortality drivers, features of health services and systems, and major historical or policy events in the African region. In this summary, “morbidity and mortality drivers” refer to conditions or events shaping health needs, “description of health services and systems” captures reported features of service delivery and system organization, and “key events” denote major historical, policy, or institutional developments influencing health systems.

## 4. Discussion

The evolution of health care systems in Africa, spanning from pre-colonial times to the SDG era, reveals a dynamic interplay of cultural, social, and political factors that shaped health practices and health care delivery [61]. This review identified 83 records on African health histories. While 18 studies provided historical overviews of Africa as a whole, only 21 country-specific historical accounts were included.

Our findings suggest that in the pre-colonial era, health care practices were deeply rooted in cultural traditions and community-driven systems. However, the colonial period introduced significant changes to this landscape. The majority of studies from this review focused on the colonial, PHC, and MDG periods, which could be attributed to several reasons. The colonial period was marked by significant transformations, including the establishment of health infrastructure, disease control programs, and the introduction of Western health care systems, which continue to shape present-day health systems. In fact, this shift was thought to result in tensions between indigenous health care systems and colonial influences [19]. Some key findings worthy of note are how records showed that, contrary to stereotypes, Africa had advanced medical and public health practices even during the pre-colonial era [2,21]. The continent played a crucial role in the discovery and treatment of diseases like malaria, trypanosomiasis, smallpox, syphilis, tuberculosis, and plague [2].

The post-independence period was marked by a renewed focus on autonomy and self-determination in health care. This period was characterized by health promotion initiatives and the eradication of diseases such as smallpox. The PHC era, influenced by the Alma-Ata Declaration (1978) [4,62], emphasized the importance of community-based health care and equity in service delivery. The focus on primary health care, maternal and child health, and the integration of community health workers into the system highlighted the importance of accessibility and community engagement in achieving health outcomes. The subsequent MDG era further expanded on targeted interventions, particularly for infectious diseases like HIV/AIDS and malaria. Finally, the SDG era currently aims to build resilient and inclusive health care systems that ensure universal health coverage.

The evidence described above may not be exhaustive for a couple of reasons. Identifying and selecting studies on African health histories was challenging due to the lack of uniformity in how histories were presented in publications. Country-specific evidence was based on 21 African countries, with countries like South Africa, Nigeria, and Ghana providing the majority of the evidence. Nonetheless, the WHO African regional office is currently looking at ways to document individual country histories. For example, a country-specific historical document from Uganda has just been published [80]. Additionally, we could not retrieve the full texts of some publications, particularly those from earlier time blocks (pre-colonial and colonial periods). Such publications only had abstracts, which did not provide sufficient evidence, subsequently resulting in their exclusion from our synthesis despite their potential eligibility. We also acknowledge that some colonial-era documents and translated materials may carry inherent biases, which could affect the completeness or accuracy of historical accounts.

Although our findings align with what is already known, we highlight certain gaps and potential limitations. The historical records available are not generalizable to all countries, as some countries’ records were not available. Therefore, more historical publications are required from individual countries. Given the high heterogeneity of the studies, we may have missed some vital records that included historical contexts. Hence, we recommend establishing a standardized guideline for reporting public health historical documents, drawing inspiration from other established guidelines for reporting studies, such as STrengthening the Reporting of OBservational studies in Epidemiology (STROBE) [96]. This would help ensure uniformity, improve comparability, strengthen the evidence base, and support the long-term use of historical public health information.

Furthermore, historical contexts should be incorporated into public health reports or documented separately. Public health professionals should collaborate with social scientists in developing such reports. There should be increased awareness within the health, public health, and broader scientific communities about the importance of including historical contexts in their publications, as these will be valuable for future research and understanding.

Funding mechanisms were not explicitly mentioned in the majority records we included, particularly regarding how users paid for the services. A possible explanation may be that funding information may not have been comprehensively or systematically documented or may have been overlooked, especially during early periods. Most studies in our review focused on health care practices, disease control, and service delivery, rather than economic aspects. Researchers might have prioritized describing these areas without detailing the financial mechanisms involved.

Given the historical nature of the information, our conclusions are based mostly on the authors’ accounts, and we did not conduct quality assessments to verify the accuracy of these accounts, especially for older historical records. In addition, the lack of standardized documentation for historical health records posed challenges for inclusion of studies in this review, potentially limiting the representativeness of our synthesis. Some articles written in other languages, such as French, were translated using Google Translate, which may have introduced slight distortions in meaning. However, we do not consider these distortions to be significant.

We acknowledge that relying primarily on PubMed presents a limitation for historical research, as it may not adequately capture historical accounts and archival sources, potentially affecting the comprehensiveness of our synthesis. Our future studies will involve gathering more data at the country level, which will greatly complement the evidence from our review. For example, a country-specific historical document from Uganda has just been published [80].

We recommend conducting primary field studies at the country level, taking a cue from the Ugandan example [80]. Also, given the perceived tensions between indigenous and colonial health care systems as identified from this review, it will be useful to have a deeper exploration of the complexity of the transition and their long-term effects. This exploration could include examining how colonial health policies shaped or displaced traditional systems as well as the social and political consequences.

Lastly, it will be useful to physically review documents that are currently only available as hard copies, especially those archived in libraries and other institutions, as well as capturing oral histories from individual countries. These oral histories could include interviews with community leaders or health care practitioners and leaders and hence could provide personal perspectives and lived experiences that are often not found in written records. Lastly, given the digital age we live in, establishing robust digital archiving practices is essential. This will ensure that these valuable histories, both oral and written, remain accessible and preserved for future reference.

## 5. Conclusions

Our scoping review of African health histories identified a reasonable amount of records across the six time blocks, from the pre-colonial period to the SDG era, depicting the diverse ways in which historical events have shaped health systems across Africa. These influences span areas such as traditional medicine, disease control, health care policies, surgical advancements, vaccine development, and financial reforms. However, the records are not exhaustive or fully representative of all countries in Africa. This necessitates the development of more country-specific historical records, the extraction of additional records from archived documents in libraries and elsewhere, and the creation of a centralized repository for future reference. By addressing these gaps, we can ensure a more comprehensive understanding of African health histories, which will help shape effective health interventions moving forward.

## Figures and Tables

**Table 1:** Health care evolution in Africa across the unique time periods *.

Period	Number of Studies Included	Morbidity and Mortality Drivers (Examples)	Description of Services and System (Examples)	Key Events (Examples)
Pre-colonial	4	Smallpox Bubonic plague	Traditional practices, led by community leaders such as chiefs and kings	Traditional healing
Colonial	13	Onchocerciasis, cholera Malaria Leptospirosis Dengue Chikungunya, Rift Valley fever Poliomyelitis Schistosomiasis	Colonial influence on health systems Indigenous/colonial strife, early surgical practices Creation of health centers and hospitals Missionary-owned hospitals	Restructuring of health systems by colonialists
Post-independence	5	Malaria	Health promotion Surgical advancements	Smallpox eradication
PHC Era	12	HIV Yellow fever Malaria	Workforce training to address shortages and improve services Training of traditional birth attendants to improve maternal and child services Scaling of maternal services Integration of traditional medicine Family planning services Prevention of mother-to-child transmission (of HIV) services Establishment of surveillance systems for infectious diseases Local production of medicines and health products and establishment of regulatory policies	Alma-Ata Declaration
MDG	12	Ebola Meningitis HIV/AIDS Malaria TB Armed conflicts	Evolution of the PHC system Establishment of public Health institutes and drug regulatory agencies Outbreak response programs Scaling up of PMTCT, HIV, and TB response programs Health financing initiatives Scaling up of vaccine introduction Private health sector evolution	MDG adoption
SDG	4	Malaria Mpox Ebola Armed conflicts COVID-19	Health financing initiatives towards SDG New community health care program Missionary and faith-based organizations role in health system strengthening Financial reforms Response to outbreaks and epidemics Scaling up of surveillance and diagnostic laboratories Expansion of malaria control efforts	SDG adoption, UHC focus

* Items listed are illustrative and do not imply hierarchy.

## Data Availability

Data generated for this review were all from publicly available publications. No new data were created or analyzed in this study.

## Appendix A

**Table A1:** Search history on PubMed: African health histories—8 September 2024.

Search	Query	Results
#4	#1 AND #2 AND #3	5170
#3	“Public health”[Title/Abstract] OR “health system”[Title/Abstract] OR “health practice”[Title/Abstract] OR “medicine”[Title/Abstract] OR “medical practice”[Title/Abstract] OR “health services”[Title/Abstract] OR “primary health care”[Title/Abstract] OR “hospital services”[Title/Abstract]	1,272,251
#2	History [Title/Abstract] OR histories [Title/Abstract] OR evolution[Title/Abstract] OR story[Title/Abstract] OR stories[Title/Abstract]	1,391,559
#1	Africa[Title/Abstract] OR Africa[Title/Abstract] OR African[Title/Abstract] OR Algeria[Title/Abstract] OR Angola[Title/Abstract] OR Benin[Title/Abstract] OR Botswana[Title/Abstract] OR “Burkina Faso”[Title/Abstract] OR Burundi[Title/Abstract] OR “Cabo Verde”[Title/Abstract] OR Cameroon[Title/Abstract] OR Cameroun[Title/Abstract] OR “Canary Islands”[Title/Abstract] OR “Cape Verde”[Title/Abstract] OR “Central African Republic”[Title/Abstract] OR Chad[Title/Abstract] OR Comoros[Title/Abstract] OR Congo[Title/Abstract] OR “Cote d’Ivoire”[Title/Abstract] OR “Democratic Republic of Congo”[Title/Abstract] OR Djibouti[Title/Abstract] OR Egypt[Title/Abstract] OR Eritrea[Title/Abstract] OR eSwatini[Title/Abstract] OR Ethiopia[Title/Abstract] OR Gabon[Title/Abstract] OR Gambia[Title/Abstract] OR Ghana[Title/Abstract] OR Guinea[Title/Abstract] OR Guinea- Bissau[Title/Abstract] OR “Ivory Coast”[Title/Abstract] OR Jamahiriya[Title/Abstract] OR Kenya[Title/Abstract] OR Lesotho[Title/Abstract] OR Liberia[Title/Abstract] OR Libya[Title/Abstract] OR Madagascar[Title/Abstract] OR Malawi[Title/Abstract] OR Mali[Title/Abstract] OR Mauritania[Title/Abstract] OR Mauritius[Title/Abstract] OR Mayotte[Title/Abstract] OR Morocco[Title/Abstract] OR Mozambique[Title/Abstract] OR Namibia[Title/Abstract] OR Niger[Title/Abstract] OR Nigeria[Title/Abstract] OR Principe[Title/Abstract] OR Reunion[Title/Abstract] OR Rwanda[Title/Abstract] OR “Saint Helena”[Title/Abstract] OR “Sao Tome”[Title/Abstract] OR Senegal[Title/Abstract] OR Seychelles[Title/Abstract] OR “Sierra Leone”[Title/Abstract] OR Somalia[Title/Abstract] OR “St Helena”[Title/Abstract] OR Sudan[Title/Abstract] OR Swaziland[Title/Abstract] OR Tanzania[Title/Abstract] OR Togo[Title/Abstract] OR Tunisia[Title/Abstract] OR Uganda[Title/Abstract] OR “Western Sahara”[Title/Abstract] OR Zaire[Title/Abstract] OR Zambia[Title/Abstract] OR Zimbabwe[Title/Abstract]	656,736
# Search number		

**Table A2:** Characteristics of included records on African health histories.

Study ID (First Author’s Last Name)	Year of Publication	History Year/Period	Time Block	Country or Sub-Region	Type of Study Design	Historical Context
Abdoulaye [45]	2015	2014	The MDG period (2000–2015)	Senegal	Narrative review	Experience of the management of the first imported Ebola virus disease case in Senegal.
Abdullahi [16]	2011	1840–1860	Colonial (1880–1959)	Africa	Narrative review	The paper explores the trends and challenges associated with traditional medicine in Africa, as well as the influence of colonialism on the evolution of African traditional medical practices.
Adetiba [20]	2022	1920–1950	Colonial (1880–1959)	Nigeria	Narrative review	A historical analysis of the role African chiefs played in the colonial health system, highlighting the tensions between colonial and indigenous health care practices.
Adusei [93]	2024	2000–2021	MDG to SDG	Ghana	Systematic review	Tracks the evolution of Community-Based Health Planning and Services (CHPS) since its inception as part of the country’s primary health care system.
Aidoo [21]	1982	1982	Colonial (1880–1959)	Ghana	Narrative review	This paper discusses some of the implications of colonialism and neo-colonialism for health care in rural areas in Ghana, highlighting the need for structural change.
Akpala [33]	1994	1975	Primary Health Care (PHC) (1970s–1990s)	Nigeria	Narrative review	Training of traditional birth attendants to improve maternal and child services.
Ario [92]	2022	2013–2021	MDG to SDG	Uganda	Narrative review	This article describes the processes followed in setting up the Uganda National Institute of Public Health (UNIPH) in 2013 and the successes and challenges encountered during the journey of its development.
Awah [34]	2015	1974	Primary Health Care (PHC) (1970s–1990s)	Uganda	Narrative review	A commentary on the controversies around the history, naming, context, and global action related to Ebola virus disease.
Baba [79]	2016	1912–2010	Colonial to MDG	Africa	review	A review of the history of Rift Valley fever outbreaks in eastern Africa to identify the epidemiological factors that could have influenced its increasing severity in humans.
Bannister [23]	2022	1909–1957	Colonial (1880–1959)	Ghana	Narrative review	This article discusses the history of the sleeping sickness and endemic onchocerciasis epidemics in colonial northern Ghana from 1909 to 1957.
Beaudevin [86]	2023	1950s–2000s	Colonial to SDG	Tanzania	Narrative review	It explores the historical development and implementation of primary health care (PHC) in Tanzania and how their experiences inform current debates on universal health coverage (UHC).
Benatar [66]	1989	1912–1989	Colonial to PHC	South Africa	Narrative review	A historical perspective on the South African health system, from the first medical training in 1912, to the establishment of the National Health Service Commission in 1940, to the challenges during and after apartheid. Emphasis was placed on addressing political and economic barriers to achieving equitable health services for all South Africans.
Benton [37]	2002	1974	Primary Health Care (PHC) (1970s–1990s)	Western Africa	Narrative review	Focuses on the historical development of a major public health partnership and its impact on the control of river blindness in Africa through the establishment of the Onchocerciasis Control Programme in West Africa (OCP).
Bernault [22]	2008	1900s	Colonial (1880–1959)	Africa		Gleaning lessons from endemic diseases, epidemics, and pandemics in Africa, this study highlights the historical involvement of Africans in biomedicine, challenging persistent misconceptions and highlighting significant contributions to global health.
Bump [24]	2022	Late 19th and early 20th century	Colonial (1880–1959)	Africa	Narrative review	This study explores the decolonization of global health through a focus on malaria and European colonialism in Africa.
Burton [72]	2015	1990–2012	PHC to MDG	South Africa	Narrative review	This article discusses the evolution of the HIV epidemic in South Africa and gives a historical overview of the struggle to establish a national PMTCT and the impact of delaying PMTCT and treatment programs on infant and maternal health.
Chipare [28]	2020	1990	Post- independence (1960–1969)	Namibia	Narrative review	This paper discusses the historical development of health promotion in Namibia.
Coghe [74]	2022	Before 20th century to colonial period	Pre-colonial to Colonial	Africa	Narrative review	The evolution of disease control and public health practices in sub-Saharan Africa, with a focus on the interactions between indigenous and colonial medical systems.
Coovadia [46]	2009	2009	The MDG period (2000–2015)	South Africa	Narrative review	Historical roots of current public health challenges in South Africa: How their painful past impedes achieving the MDG. Challenges including historical policies from colonialism, apartheid, and the post-apartheid period, including racial and gender discrimination, the migrant labor system, income inequality, extreme violence, economic disparities, leadership and management failures, primary health care deficiencies, human resources crisis, and the impact of the HIV epidemic
David [61]	2020	1900s–1980s	Colonial to PHC	Central Africa	Archival research	Focuses on a historical perspective on the evolution of surveillance systems in the Central African Republic and its impact on the broader health system, particularly on how surveillance has been prioritized over primary health care.
Dembek [49]	2024	20th century	The MDG period (2000–2015)	Africa	Descriptive study	This review discusses important aspects of notable Ebola outbreaks in Africa and public health responses and impacts on disease severity and spread.
Digby [80]	2008	1900s–2007	Colonial to MDG	South Africa	Narrative review	Provides a wide-ranging historical analysis of South African medicine, covering key aspects of the health care system, public health, and the influence of both colonial and post-colonial contexts.
Diop [25]	2021	1879	Colonial (1880–1959)	Uganda	Narrative review	Discusses advanced surgical and anesthesia practices in Bunyoro (present-day Uganda) in 1879, demonstrating that their practices were well-developed compared to other sub-Saharan African countries and even Western medicine of that time. Challenging stereotypes.
Doyle [26]	2022	1940s–1950s	Colonial (1880–1959)	Africa	Narrative review	Insights into the historical context of health and disease in Africa, covering key periods that have shaped current health systems.
Ericksen-Pereira [29]	2018	1960s	Post-independence (1960–1969)	South Africa	Qualitative design	This article explores the history of naturopathy in South Africa.
Feierman [27]	1985	1900s	Colonial (1880–1959)	Africa	Narrative review	This paper examines the social determinants of health and health care in Africa over the past century, suggesting that healers—whether doctors or traditional practitioners—have been less influential in shaping health outcomes than is commonly believed.
Fenwick [81]	2006	1918–2006	Colonial to MDG	Africa	Narrative review	Focuses on the history of and strategies for controlling schistosomiasis.
Foege [30]	1975	1966–1970s	Post-independence (1960–1969)	West Africa and Central Africa	Narrative review	A historical perspective on one of the most significant public health achievements in West and Central Africa—the eradication of smallpox and control of measles—and the impact this had on the development of health systems in the region.
Gaber [82]	2013	1893–2013	Colonial to MDG	Ivory Coast (Côte d’Ivoire)	Systematic review	Examines the impact of historical factors, such as armed conflicts in former French colonies, particularly Côte d’Ivoire, on health systems. The study highlights how these conflicts exacerbated historically inherited challenges, including the unequal distribution of health services, a bias toward urban curative care, inadequate human resources, and weak health governance.
Gaüzère [75]	2013	1729–1930s	Pre-colonial to Colonial	Madagascar	Narrative review	A comprehensive historical account of a wide range of the major infectious diseases that have affected Madagascar and the surrounding islands over the past few centuries, such as smallpox, cholera, Bubonic plague, Leptospirosis, Dengue, Chikungunya, Rift Valley fever, and HIV/AIDS, amongst others. Includes public health interventions, vaccine development, and the establishment of the Pasteur Institute.
Gorsky [62]	2023	1951–1985	Colonial to PHC	Africa		This article explores the prior historical context to today’s debates about extending ‘health for all’ in Africa.
Grieve [59]	2018	2018	The SDG period (post 2015)	Ghana	historically focused mixed methods	The evolution and contribution of faith-based non-profit providers to the development of the Ghanaian health system in advancing towards universal health coverage.
Harries [63]	2013	1800–1990s	Colonial to PHC	Africa	Narrative review	A report on how missionary societies contributed to the development of health systems in three African countries: South Africa, Ghana, and Tanzania.
Havik [15]	2014	1945	Colonial (1880–1959)	Guinea	Narrative review	This paper describes how the Portuguese Guinean colonial government did not prioritize health services until the creation of the Commission for the Study and Combat against Sleeping Sickness in 1945. Despite poor funding and staffing, the commission successfully controlled endemic diseases and provided free preventative medical care, reducing the spread of Human African Trypanosomiasis.
van Rensburg [50]	2014	2014	The MDG period (2000–2015)	South Africa	Narrative review	Focuses on the history and post-apartheid reforms of South Africa’s Human Resources for Health, focusing on workforce shortages, unequal distribution, colonial policies, and health system reforms like the National Health Insurance Plan.
Kagaayi [38]	2016	1983	Primary Health Care (PHC) (1970s–1990s)	Africa	Narrative review	The history of the HIV/AIDS epidemic in Africa.
Kalua [51]	2017	2002	The MDG period (2000–2015)	Malawi	Narrative review	Focuses on the evolution of preventing mother-to-child transmission of HIV through the development and implementation of a new policy (Option B+ strategy—test and start treatment) in Malawi. Its impact on the country’s health system and the lessons learned during its rollout, with insights from Cameroon and Tanzania, are highlighted.
Karema [87]	2020	1900 and 2018	Colonial to SDG	Rwanda	Systematic review	A comprehensive review of the evolution of malaria epidemiology and control in Rwanda is presented to help understand the potential drivers of change and to inform the future of the fight against malaria in the country.
Karita [39]	2016	1988–1994	Primary Health Care (PHC) (1970s–1990s)	Rwanda	Narrative review	This article reports the successful nationwide implementation of couples’ voluntary HIV counseling and testing (CVCT) in Rwanda.
Kautzky [64]	2008	1940–1994	Colonial to PHC	South Africa	Narrative review	The evolution of the PHC in South Africa from its onset to different reforms, coupled with challenges faced and future considerations.
Kwiecinski [12]	2013	3000 BCE (before common era)	Pre-colonial (before or by 1879)	Egypt	Narrative review	Attempts to describe the history of medicine in Egypt by linking to the biography of a physician-king known as “Aha”.
LeVasseur [69]	2014	1980s–2014	PHC to MDG	Africa	Narrative review	History of HIV epidemic in Africa, trajectory of epidemiology, and public health response.
Mabika [67]	2015	1900s-post 1994	Colonial to PHC	Southern Africa	Narrative review	The study explores how Swiss medical doctors introduced public and community health practices in north-eastern South Africa; a non-colonial perspective on health care development. It highlights how Swiss missions exploited local authorities’ neglect to implement their own model of care, challenging segregationist policies and aligning with anti-apartheid efforts.
Mabunda [40]	2023	1977, 1978, 1995	Primary Health Care (PHC) (1970s–1990s)	South Africa	(a) A literature review, (b) a policy review, and (c) semi-structured interviews	Training of health workforce—history of design and implementation of the Return of service (RoS) scheme in three Southern African countries, Botswana, Eswatini, and Lesotho—a loan scheme that addressed the gap in human resources in health care to enhance employment opportunities for citizens, develop a public sector workforce that meets global competency standards, and support the career advancement of government employees.
Makwero [52]	2017	2001–2015	The MDG period (2000–2015)	Malawi	Narrative review	Provides a historical overview of the development of family medicine training and practice in Malawi, tracing its origins and evolution.
Manton [32]	2018	1960s	Post-independence (1960–1969)	West Africa and Central Africa	Narrative review	This article examines the 1960s WHO-USAID national health planning efforts in West and Central Africa, highlighting challenges faced in administrative capacity, financial resources, and implementation.
Muga [89]	2010	1965–1997	Post-independence to PHC	Kenya	Document analysis	This paper analyzes the evolution of mental health policy in Kenya during the period 1965–1997.
Muhirwe [31]	2006	1962	Post- independence (1960–1969)	Uganda	Narrative review	The paper looks into the evolution of dentistry in Uganda as well as the havoc wrecked on health care in general by two decades of civil strife in an effort to find explanations for the poor performance of the oral health system.
Mukanga [41]	2011	1990s	Primary Health Care (PHC) (1970s–1990s)	Africa	Descriptive study	Training- Establishment of the African Field Epidemiology Network (FETP) to address critical shortages of epidemiologists and public health workforce
Mureithi [53]	2018	2013	The MDG period (2000–2015)	South Africa	Multi-case study	This paper explores the early inception and emergence of the general practitioner contracting initiative (GPCI) as a health financing initiative for national health insurance achieving universal health coverage
Myers [42]	1989	1970s	Primary Health Care (PHC) (1970s–1990s)	South Africa	Narrative review	This paper seeks to present an account of the occupational health situation in South Africa
Nimo [90]	1984	1960–1984	Post- independence to PHC	Ghana	Narrative review	An account of the evolution of Ghana’s health manpower system and its alignment with the country’s primary health care strategy. The study highlights how, since independence, the country focused on training health workers to meet local needs, particularly in rural areas, and outlines the three-tier health system that was introduced to manage the delivery of primary health care services.
Nott [17]	2019	1900s	Colonial (1880–1959)	Africa	Narrative review	The historical context in the 20th century and political (British colonial) influences on nutritional health practices and policies in Africa. Specifically, the belief that African diets were severely deficient in protein, leading to widespread childhood protein deficiency and contributing to African economic underdevelopment.
Nsanzimana [43]	2015	1983–1994	Primary Health Care (PHC) (1970s–1990s)	Rwanda	Historical analysis	The historical analysis of Rwanda’s health system development 20 years post-genocide, particularly focusing on HIV treatment and its broader impact on the health system
Odutolu [73]	2016	1986–2015	PHC to MDG	Nigeria	Descriptive study	This case study aims to describe the evolution of the primary health care system in Nigeria from the primary care era to sustainable development goals (SDG) era, specifically, the Primary Health Care Under One Roof (PHCUOR) policy as part of the broader PHC reforms.
Olumade [77]	2020	1864–2020	Pre-colonial to SDG	Nigeria	Historical overview	Provides historical overview of the challenges and successes of disease outbreak responses in Nigeria such as Lassa fever, Ebola virus disease, Yellow fever disease, Poliomyelitis, mpox
Oppong [44]	1989	1989	Primary Health Care (PHC) (1970s–1990s)	Nigeria	Narrative review	The role of traditional medicine in African health systems and the evolving relationship between traditional and Western medical practices
Ouoba [18]	2022	1895–1960	Colonial (1880–1959)	Western Africa	Narrative review	This history focuses on the how France had to balance colonization and health promotion. This impacted on the role of pharmacists which were multidisciplinary and it is thought that the current roles of pharmacists in Francophone West Africa are inherited from colonial pharmacy practices.
Patel [54]	2019	2014	The MDG period (2000–2015)	Western Africa	Narrative review	The history of the MenAfriNet consortium which aims to support the strategic implementation of case-based meningitis surveillance in five key countries: Burkina Faso, Chad, Mali, Niger, and Togo.
Petu [57]	2018	2018	The SDG period (post 2015)	Africa	Narrative review	The paper tells the story of importance of planning using the national comprehensive multi-year strategic plans (cMYP) processes to immunization financing sustainability as a necessary condition in the trajectory towards sustainability.
Pfeiffer [58]	2017	2017	The SDG period (post 2015)	Mozambique	Narrative review	This study looks at how IMF’s austerity measures affected Mozambique’s health system, focusing on the challenges of coordinating foreign aid to strengthen the health system while dealing with limited government funding. And impact on achieving sustainable development goals
Phillips [83]	2014	1940s and 1990s and 2014	Colonial to MDG	South Africa	Narrative review	Highlights the historical evolution and impact of a primary health care model, Pholela experiment in South Africa
Planned Parenthood Federation of America Family Planning International Assistance United States Agency for International Development [68]	1977	1950s–1970s	Colonial to PHC	Kenya	Narrative review	It provides historical context regarding the evolution of integrated maternal health and family planning programs in Kenya, highlighting the role of local self-help initiatives (“harambee”) and international support in expanding these services.
Pourraz [91]	2022	1960s- date	Post- independence to SDG	Ghana	Narrative review	This study focused on how Ghana’s dependence on medicine imports has evolved since independence and examines the country’s efforts and challenges in promoting local pharmaceutical production over time.
Said [55]	2024	21st century	The MDG period (2000–2015)	Somalia	Narrative review	This paper explores the current health care landscape in Somalia, highlighting the challenges faced by health care systems under federal governance. It also examines the historical development of Somalia’s health care system and the introduction of new federalist principles, providing a comprehensive analysis of health care governance, financing, and historical evolution in the country.
Schewitz [19]	2022	1940s	Colonial (1880–1959)	South Africa	Narrative review	The development and evolution of cardiothoracic surgery in South Africa, starting in Cape Town and gradually expanding to the rest of the country and, eventually, to other sub-Saharan African countries, particularly after the end of apartheid.
Scott-Emuakpor [84]	2013	1945–2008	Colonial to MDG	Nigeria		Describes how the health care system evolved in Nigeria from the colonial days to the new millennium, including policies and health workforce disease control and response.
Shamlaye [56]	2020	20th century	The MDG period (2000–2015)	Seychelles	Narrative review	An overview of health in Seychelles.
Simelela [71]	2014	1980s–2014	PHC to MDG	South Africa	Narrative review	Challenges in the HIV response and how civil society played a role in the response to HIV after years of denialism by the government.
Simon [70]	2009	1970–2007	PHC to MDG	Mozambique	Observational study	This paper provides a participant-observer perspective of the evolution of community health workers from vertical and isolated activities for TB, HIV, and other specific diseases to an integrated community health team approach for tackling the main disease burden in a rural district of Mozambique.
Soumonni [13]	2012	Nineteenth century	Pre-colonial (before or by 1879)	Benin	Narrative review	The essay examines, with special reference to smallpox, the perception and interpretation of disease in pre-colonial Dahomey, which in the present day is the Republic of Benin.
Tiffay [47]	2015	2001	The MDG period (2000–2015)	Africa	Narrative review	The implementation of the Meningitis Vaccine Project (MVP) which aimed to develop, test, license, and introduce a group A meningococcal (MenA) conjugate vaccine specifically for sub-Saharan Africa.
Tornimbene [48]	2022	2015	The MDG period (2000–2015)	Africa	Narrative review	The article outlines the progress made in Africa with the implementation of the Global Antimicrobial Resistance and Use Surveillance System (GLASS) reporting by African countries; development of national AMR surveillance systems; and the reporting of AMR data by African countries from 2017 to 2019, highlighting the challenges, perceived impacts, and benefits of participating in GLASS.
United Nations. Department of International Economic and Social Affairs. Population Division, and United Nations Fund for Population Activities UNFPA [35]	1985	1985	Primary Health Care (PHC) (1970s–1990s)	Eswatini	Narrative review	Focuses on government efforts to address population issues through the implementation of family planning services to control birth rates, among other socio-economic interventions.
United Nations. Department of International Economic and Social Affairs. Population Division, and United Nations Fund for Population Activities UNFPA [36]	1984	1984	Primary Health Care (PHC) (1970s–1990s)	Ethiopia	Narrative review	The paper discusses the government’s approach to health and population issues, highlighting concerns over high fertility and mortality rates, as well as an emphasis on improving health through primary health care.
van den Heever [85]	2012	1889–1999 and 2000–2010	Colonial to MDG	South Africa	Narrative review	It examines how the private health sector has complemented the public health system in South Africa and evolved over the years, contributing to universal coverage, particularly in the context of health care financing.
van der Walt [65]	2002	1935–1975	Colonial to PHC	South Africa	Narrative review	The authors explore both the explicit and more functional reasons for maintaining task orientation, as well as the implicit and mostly unconscious socially structured defenses which contribute to the continuation of this form of practice.
Vargas [14]	2012	3rd to 4th millennium BC	Pre-colonial (before or by 1879)	Egypt	Narrative review	It provides insight into how medical knowledge evolved in ancient Egypt, citing the Edwin Smith papyrus (PES), and how it is similar to modern clinical methods. Therefore, it suggests that the roots of medicine can be traced back to ancient Egypt.
Waite [2]	1987	Before 20th century	Pre-colonial (before or by 1879)	East Africa and Central Africa	Narrative review	The study focuses on the history of public health in Africa before the 20th century, specifically on traditional African practices like rainmaking and sorcery control, highlighting the control exercised by kings, chiefs, and priests in regions such as South Eastern Zaire, southern Tanzania, Zambia, Malawi, northern Mozambique, and Zimbabwe.
Walker [76]	2001	1876–2001	Pre-colonial to MDG	South Africa	Narrative review	The historical analysis of the public health landscape in South Africa, highlighting important trends in mortality, life expectancy, and health challenges over time. Includes the impact of social and environmental factors, as well as the significant role of HIV/AIDS.
Whyle [88]	2023	1920s–2019	Colonial to SDG	South Africa	Literature review	The review covers policy processes from the 1920s to 2019, specifically describing the evolution of health financing reforms and the impact of socio-political factors on reform efforts at different time points
World Health Organization African Region [78]	2023	150 years	Pre-colonial to SDG	Uganda	Report	This report provides an overview of all historical events that occurred in each time block between the pre-colonial to SDG periods in Uganda.
Wintrup [60]	2023	2019–2020	The SDG period (post 2015)	Zambia	Narrative review	The assessment of a new community health care worker program which was initially developed as part of the Alma Ata; challenges faced by the program and how it played a role in strengthening health systems and helping to achieve universal health coverage.
